# Germany’s Epidemiological Data on Cleft Lip and Palate After the Chernobyl Nuclear Power Plant Accident: A Systematic Literature Review

**DOI:** 10.3390/epidemiologia7050123

**Published:** 2026-09-01

**Authors:** Claas Willem Molsen, Pascal Grün, Christian Philipp Schnell, Flora Turhani, Sebastian Fitzek, Marius Meier, Dritan Turhani

**Affiliations:** 1 Center for Oral and Maxillofacial Surgery, Department of Dentistry, Faculty of Medicine and Dentistry, Danube Private University, Steiner Landstrasse 124, 3500 Krems an der Donau, Austria; 2 Clinical Application of Artificial Intelligence in Dentistry (CAAID) Group, Department of Dentistry, Faculty of Medicine and Dentistry, Danube Private University, Steiner Landstraße 124, 3500 Krems an der Donau, Austria; 3 Health Services Research Group, Medical Images Analysis and Artificial Intelligence (MIAAI), Department of Medicine, Faculty of Medicine and Dentistry, Danube Private University, Steiner Landstrasse 124, 3500 Krems an der Donau, Austria; 4 Dental Practice, 9490 Vaduz, Liechtenstein

**Keywords:** systematic review, Chernobyl, Germany, epidemiology, cleft lip and palate, malformations

## Abstract

Background/Objectives: The aetiology of facial clefts remains incompletely understood, and ionising radiation has been discussed as a potential exogenous factor. This review investigated the epidemiology of cleft lip and/or palate (CL/P) in Germany in relation to the Chernobyl NPP accident. Methods: A systematic literature search was conducted using MEDLINE, LIVIVO, and the Cochrane Library, supplemented by additional searches. OHAT risk-of-bias domains were applied to the included studies. Results: In total, 276 potentially relevant records were identified, of which six were included. Studies from Bavaria and the former German Democratic Republic reported regional associations between increased CL/P prevalence and the Chernobyl NPP accident. None of the included studies assessed radiation exposure at the individual level; exposure assessment was regional or ecological. Conclusions: Associations were reported in some more heavily contaminated regions, whereas no statistically significant differences were found in regions affected by lower levels of fallout. One study reported an estimated additional regional exposure of 1.2 mGy in the most heavily contaminated areas. This estimate does not substitute for individual dosimetry and should be interpreted cautiously. Experimental and other sources cited in the review discuss malformation effects at doses of at least 50 mGy. Given the heterogeneous study designs, ecological exposure assessment, inadequate control of confounding, and very low certainty of evidence, the available data do not support establishing a causal relationship between Chernobyl-related exposure and CL/P in Germany.

## 1. Introduction

Many official statements have been issued concerning the consequences of the Chernobyl Nuclear Power Plant (NPP) accident in the context of congenital malformations. However, the sources of information in these statements remain unclear. This also applies to Germany, where a debate over this topic continues to this day. Literature pertaining to the prevalence of cleft lip and/or palate (CL/P)—or more specifically, the various subtypes thereof—remains especially scant. These subtypes include cleft lip (CL), cleft palate (CP), and cleft lip and palate (CLP). This review aims to provide reliable information on the impact of the disaster on the prevalence of CL/P.

German governmental bodies have generally assessed the likelihood of observable health consequences of the Chernobyl accident in Germany as low [1,2]. Other organisations, including the International Physicians for the Prevention of Nuclear War (IPPNW), have published alternative assessments of the health consequences of Chernobyl [3]. These sources differ in scope, methodology, and publication context. The present review does not seek to adjudicate between institutional positions, but to examine the epidemiological literature specifically concerning CL/P in Germany.

The public discussion surrounding nuclear power and its health consequences remains politically charged. Between 2024 and 2025, the German Federal Office for Radiation Protection (BfS) website changed: a paragraph reporting no adverse effects among the children of 5000 pregnant women living in more highly contaminated regions of Germany was present in an archived version but was no longer present in the later version [2,4]. The reason for this change is not inferred in the present review.

Against this background, a recent study reported an association between hospital discharges after neoplasia treatment and regional Cs-137 fallout [5]. An evaluation of the EUROCAT network of population-based congenital anomaly registries in Western Europe found no significant overall change in malformation rates, but did not specifically analyse the German CL/P data considered in the present review [6]. Other studies examined health consequences in Germany without focusing on CL/P [7,8,9].

Previous reviews have investigated malformations (and cancer) in Europe more broadly; however, no mention has been made of CL/P in Germany [10,11], and a comprehensive nationwide analysis of CL/P remains lacking.

The scope of the present study was structured according to the population, exposure, and outcomes (PEO) framework. The population under study was from the current Federal Republic of Germany (FRG), which was formerly divided into the German Democratic Republic (GDR) and FRG. In the aftermath of the Chernobyl NPP accident, the German population experienced varying levels of exposure to ionising radiation, depending on their geographical location. This study examined the extent to which this exposure changed the epidemiological parameters of CL/P in Eastern and Western Germany.

## 2. Materials and Methods

The PRISMA 2020 statement and checklist Table S1 were used as the overarching reporting framework wherever applicable [12]. This systematic review was retrospectively registered on the Open Science Framework (OSF). The corresponding DOI is: 10.17605/OSF.IO/VYX9T.

### 2.1. Eligibility Criteria

Based on the established working hypothesis, studies were eligible if they were epidemiological studies of facial clefts (excluding rare cleft types), were conducted within the current borders of Germany, and included an observation period spanning the Chernobyl accident, with data from before and after 1986 and 1986 itself included in the study window. Cohort, cross-sectional, and ecological studies were eligible. Studies reporting CL, CP, CLP, or the combined category CL/P were considered.

Studies were excluded if they examined outcomes other than facial clefts in relation to ionising radiation or Chernobyl; if the observation period ended before November 1986; if the study population was located outside the current borders of Germany; or if they were in vitro, in situ, or animal studies. Unpublished manuscripts, conference papers, and official statements were excluded, with the exception of doctoral dissertations identified through the library search that met all other eligibility criteria.

### 2.2. Information Sources and Search Strategy

The literature review was conducted within a specified timeframe, from November to December 2025, and the final verification occurred on 24 December 2025. Preliminary research was conducted to identify the key study articles. The subsequent systematic literature review was validated using articles identified in the preliminary research, which, by necessity, were incorporated into the results. To enhance the list of Medical Subject Headings (MeSH) terms, preliminary research was conducted to identify MeSH terms connected to potentially relevant studies. In addition, the MeSH database provided by the National Institutes of Health (NIH) was screened for relevant MeSH terms. The following search string was created for the PubMed electronic database (www.pubmed.gov; accessed on 24 December 2025):

((“Cleft Lip/epidemiology”[Mesh] OR “Cleft Palate/epidemiology”[Mesh] OR “Cleft Palate”[Mesh] OR “Cleft Lip”[Mesh]) AND (“Germany”[Mesh] OR “Germany, West”[Mesh] OR “Germany, East”[Mesh]) AND (“Cross-Sectional Studies”[Mesh] OR “Incidence”[Mesh] OR “Prevalence”[Mesh])) OR (“Radiation, Ionizing”[Mesh] AND “Environmental Exposure”[Mesh] AND “Chernobyl Nuclear Accident”[Mesh])

The string was adapted to the search requirements of each platform and additionally applied in the Cochrane Library (URL: www.cochranelibrary.com/central; accessed on 24 December 2025) and LIVIVO (URL: www.livivo.de; accessed on 24 December 2025). The LIVIVO search was limited to the subject area “Medicine, Health”.

(“Cleft Lip/epidemiology” OR “Cleft Palate/epidemiology” OR “Cleft Lip” OR “Cleft Palate” OR cleft*) AND (“Germany” OR “Germany, West” OR “Germany, East” OR Deutschland) AND (“Cross-Sectional Studies” OR “Incidence” OR “Prevalence”)

The electronic database search was supplemented by a library search of scientific papers at Ruhr University Bochum, Germany, and by backward citation searching of the reference lists of included studies. To obtain a broader overview of the field, the BfS (https://www.bfs.de/EN/home/home_node.html, accessed on 24 December 2025) and scientists working in the field were also consulted. No restrictions were imposed on access, full text, or language.

A systematic search of grey literature was not conducted. Two doctoral dissertations identified through the library search were retained because they met the substantive eligibility criteria and provided relevant epidemiological data. Because dissertations are grey literature, this represents an explicit exception to the general search strategy. Both dissertations were appraised using the same risk-of-bias and certainty framework as the other included studies.

### 2.3. Selection and Data Collection Process

**Figure 1:**
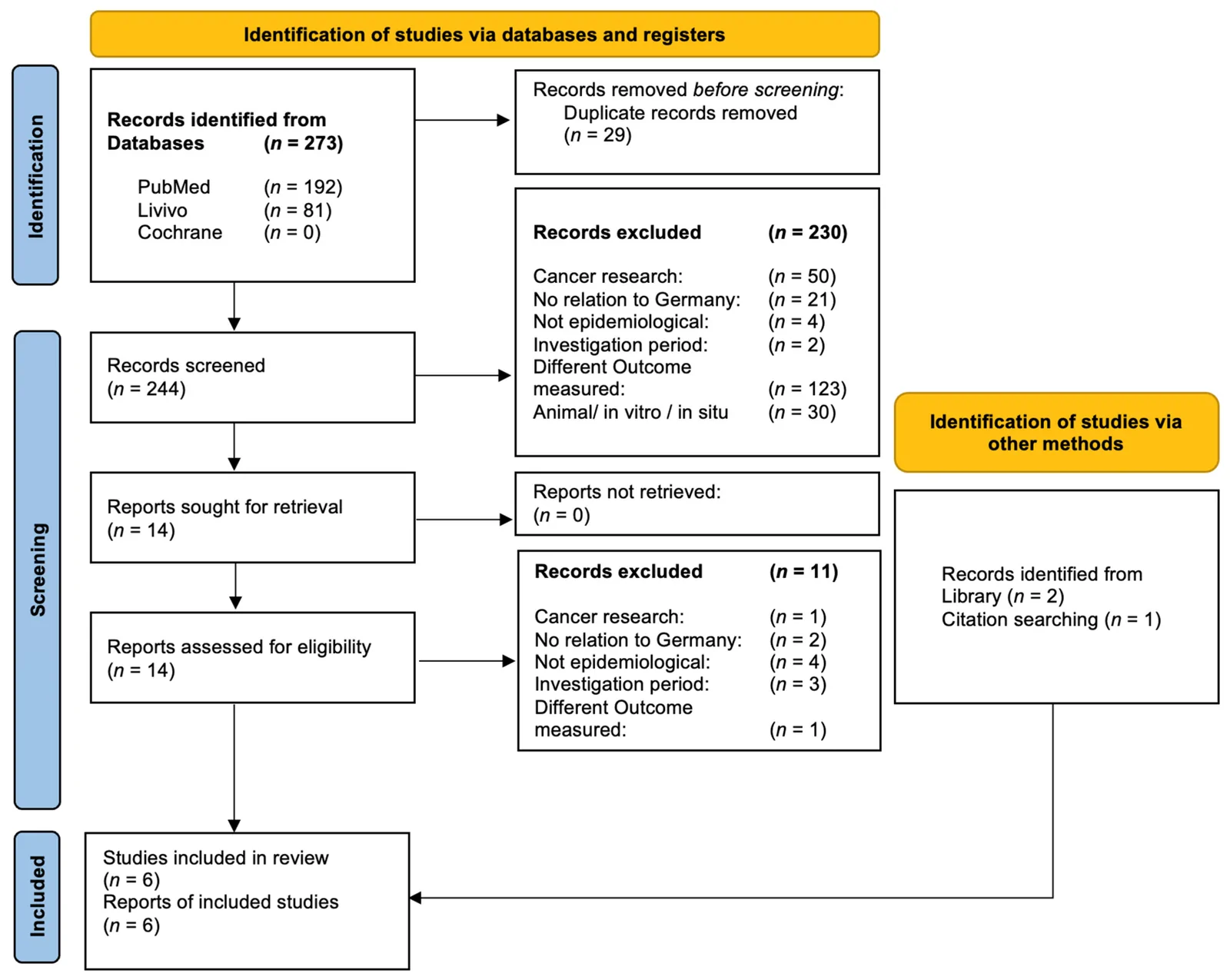
PRISMA flow diagram. According to [12].

Titles and abstracts were screened independently by two investigators (CWM and CPS). Disagreements during screening were resolved by consultation with a third investigator (DT). After full-text inclusion, the six reports were distributed between CWM and CPS for detailed review, with DT resolving disagreements. The initial allocation took the reported direction and statistical significance of findings into account to reduce confirmation bias; however, statistical significance was recorded descriptively and was not used as a criterion for evidence weighting or as the primary synthesis framework. The entire selection process is illustrated using a PRISMA flow chart (Figure 1). Zotero (version 7.0.30) was used for reference management.

Data collection was conducted by two independent study investigators (CWM and CPS). In the event of a discrepancy, a third investigator (DT) was tasked with resolving the issue. Data collection was performed manually without an automation tool. Upon screening studies relevant to the topic, they were categorised into studies reviewing the impact of Chernobyl on the prevalence of CL/P in Germany or (ii) studies reviewing the prevalence of CL/P independently of Chernobyl. Whenever the relevant information necessary for a comprehensive evaluation of the working hypothesis was not mentioned in the included study articles, the corresponding authors were contacted.

### 2.4. Data Items

The primary outcome was change in the prevalence of CL/P during observation periods spanning the Chernobyl accident, interpreted in relation to the exposure information available for each study. For each report, the direction of change, reported statistical significance when available, and the presence or absence of contamination or exposure information were extracted. These significance categories were used descriptively and not as the basis for evidence weighting or causal inference.

Other variables for which data were sought included population size, investigated region, and data origin, such as maternity units or oral and maxillofacial surgery wards, methodology, and dosimetry (first and foremost, decay rate of Cs-137). Conversely, in cases where no dosimetry was employed, a comparison was made between the survey data and the information provided by the BfS.

To ascertain the potential causality between the Chernobyl NPP accident and the prevalence of CL/P in Germany, the Bradford Hill criteria were utilised as a methodological framework, with the following criteria taken into consideration: strength, consistency, specificity, temporality, biological gradient, plausibility, coherence, experiment, and analogy.

### 2.5. Data Synthesis

The authors assessed whether quantitative pooling of the included studies would be methodologically appropriate. A formal meta-analysis was not performed because the included reports were not sufficiently comparable with respect to exposure definition, outcome operationalisation, denominator structure, study design, independence of study populations, and availability of effect estimates with measures of precision. Exposure was assessed at different ecological levels or not measured at all; outcomes were reported variably as CL/P, CLP, CP, or mixed phenotypes; and several studies did not provide adjusted effect estimates or reconstructible variance measures. Therefore, a structured synthesis without meta-analysis was considered more appropriate. Studies were grouped according to exposure ascertainment, outcome definition, denominator validity, direction of association, risk of bias, and certainty of evidence. The significance-based four-field table was retained solely as a descriptive orientation and was not used for vote counting, evidence weighting, or causal inference. The structured synthesis was complemented by a Bradford Hill-informed causal appraisal.

### 2.6. Study Risk of Bias Assessment

The Office of Health Assessment and Translation (OHAT) tool was used to evaluate the risk of bias [13]. This tool is suitable for methodologically older studies and can be applied to heterogeneous study designs. The OHAT approach for human observational studies uses a seven-question format to evaluate six distinct domains.

- Selection bias: Did selection of study participants result in appropriate comparison groups?
- Confounding bias: Did the study design or analysis account for the important confounding and modifying variables? The multifactorial aetiology of facial clefts, as outlined in the pertinent literature and including the numerous external factors under discussion, should be considered in this context. These included socioeconomic status, maternal age, folic acid intake, smoking, alcohol consumption, diabetes, and pharmaceutical use.
- Attrition/exclusion bias: Were the outcome data complete, without attrition or exclusion from the analysis? Depending on the provenance of the data, it is possible that parents with children with CL/P relocated to a different home outside or inside the area of investigation. This results in either the occurrence or absence of duplicate counting, thereby inducing an alteration in prevalence.
- Detection bias: Can we be confident in exposure characterisation and outcome assessment? The evaluation is predominantly contingent on the research design employed and the dosimetry measurement system.
- Selective reporting bias: Were all measured outcomes reported?
- Other sources of bias: Were there any other potential threats to internal validity? This assessment was conducted with particular attention paid to inadequate statistical methods, funding, and conflicts of interest.

The risk of bias was assessed by two independent investigators (CWM and CPS). Each OHAT domain was rated as definitely low, probably low, probably high, or definitely high risk of bias. When the available information was insufficient or not reported, the domain was designated as probably high (NR = not reported). Disagreements were resolved by a third investigator (DT). For descriptive study-level summarisation, the authors used an adapted numerical aggregation based on the OHAT domain ratings, assigning values from 1 to 4, from definitely low to definitely high risk of bias. An average of <1.5 was classified as definitely low, 1.5 to <2.5 as probably low, 2.5 to <3.5 as probably high, and >3.5 as definitely high risk of bias. This arithmetic aggregation is an author-defined adaptation and is not the standard OHAT tiering procedure; the domain-level ratings are therefore reported separately and should remain the primary basis for interpretation.

### 2.7. Certainty Assessment

The Grading of Recommendations Assessment, Development, and Evaluation (GRADE) approach was used to evaluate outcome certainty [14]. Along with the assessment of bias conducted in accordance with the OHAT tool, consideration was given to the presence of inconsistency, indirectness, imprecision, and publication bias. As in the case of risk of bias, a certainty assessment was performed manually by two study investigators (CWM and CPS). In cases of disagreement, a third study investigator (DT) was involved.

## 3. Results

### 3.1. Study Selection

A total of 276 potentially relevant records were identified: 273 from online databases, 2 from the Ruhr University Bochum library, and 1 through backward citation searching. Following removal of 29 duplicate database records, 244 database records were screened. Fourteen reports from the database search were sought and assessed for eligibility. Eleven were excluded because they considered periods after 1986 [15,16,17], examined regions beyond Germany [18,19], lacked epidemiological relevance [20,21,22,23], did not investigate CL/P [24], or examined radon rather than Chernobyl-related exposure [25]. Three articles from the database searches [26,27,28], one article identified through citation searching [29], and two doctoral dissertations from the library search [30,31] were included in the final analysis. The methodological selection of the studies is illustrated in the PRISMA flow chart (Figure 1).

### 3.2. Study Characteristics

Table 1 provides a descriptive orientation of the reported post-Chernobyl changes in CL/P prevalence according to broad regional contamination categories. It is not used for evidence weighting or significance-based synthesis. The structured synthesis considers exposure ascertainment, outcome definition, denominator validity, study design, risk of bias, and certainty of evidence. The regions designated as more severely contaminated are located in southern Bavaria and parts of the former GDR, particularly in the northern region; the remaining regions of the FRG are classified as less severely contaminated for this descriptive table.

It should be acknowledged that none of the existing studies have investigated these areas in isolation.

Rösche et al. [26], Zieglowski and Hemprich [27], and Scherb and Weigelt [28] analysed the prevalence of CL/P in Bavaria and the GDR. All these studies found a significant increase in the prevalence of CL/P. Irl et al. [29] concluded that there was no statistically significant difference in prevalence rates between southern and northern Bavaria. Petersen [30] conducted a study on the number of patients with CL/P treated in a German hospital in the city of Bremen. No significant increase in the number of patients was observed following the Chernobyl disaster. However, an increase in the number of patients was observed among men between 1986 and 1997, and among women between 1984 and 1998. Schlemme [31] collected data on children born in the Ruhr region. No significant changes in the prevalence were observed. Between 1984 and 1993, neither an increase nor decrease in the incidence of malformations was observed.

### 3.3. Risk of Bias in Studies

The risk of bias for each study was classified according to OHAT (Table 2). Three articles from the included studies were identified as demonstrating a probably low overall risk, while three articles were categorised as probably high.

Selection bias was considered probably high when indirect evidence suggested that the number of children born with CL/P could have been large enough to influence the outcomes. When data were derived from an oral and maxillofacial surgery unit or a non-official register, the risk of bias was assessed as probably low. Data from official registers, which were available only in the former GDR, were classified as definitely low risk. Although several studies noted potential confounding factors, these were not evaluated or adjusted for; consequently, confounding bias was assessed as probably high.

The attrition/exclusion bias was defined as definitely low. The diagnosis of CL/P is typically made within the first few days after birth, except for submucous cleft palates. Therefore, it was hypothesised that no extant bias significantly influenced the results. When data were not derived from birth registers or maternity wards, the risk was assessed at a higher level. Treatment of CL/P is not regionally bound. Consequently, the exclusion of children was possible.

Detection bias was assessed using two questions. Because both exposure characterisation and outcome assessment influence the final judgement, the more conservative rating was used. Irl et al. [29] used dosimetry, but the exposure categorisation was imprecise; consequently, detection bias was judged as definitely high. Petersen [30], Rösche et al. [26], and Schlemme [31] did not use dosimetry. Detection bias was not assessed for Petersen [30] and Schlemme [31] with respect to Chernobyl-specific exposure, because these studies were not designed to estimate the effect of ionising radiation or the Chernobyl accident. Zieglowski and Hemprich [27] used exposure information, but the report was not sufficiently precise; consequently, detection bias was assessed as probably high.

Selective reporting has been identified on several occasions. In some cases, the absolute numerical values were not specified. Occasionally, the *p*-value was not included if there was one at all and sometimes the confidence interval was not specified. The standard deviation was rarely mentioned.

It is imperative to acknowledge the significance of other potential sources of bias. None of the authors have any reference to a potential conflict of interest, nor do they provide any information regarding the financial sources for their studies. Two studies investigated the prevalence of CL/P without considering the Chernobyl disaster. Consequently, bias in these studies was categorised as definitely low.

### 3.4. Results of Individual Studies

The individual results of each included study are summarised in Table 3, Table 4 and Table 5. Table 3 summarises the relevant studies, Table 4 provides information on statistical methods, exposure measurements, and comparators, and Table 5 compares the number of cases before and after the Chernobyl NPP accident. The period and location of investigation, methodology, key outcomes relevant to the research question, and missing data or information were specified. Table 3, Table 4 and Table 5 are arranged in descending order of exposure measurement quality. Studies without dosimetry or exposure measurement [26,30,31] appear at the end of the table.

Of the studies that examine the subtypes, only two take exposure into consideration. Both consider CLP and CP separately [26,29]. One further acknowledges ionising radiation but does not consider it in its statistical work [27]. Refs. [30,31] examine subtypes in even greater depth. However, cases of CL are not regarded in this review, as none of the studies examining exposure give consideration to their prevalence.

In addition, where specified, the syndromic cases are to be distinguished from the non-syndromic ones. The extant literature on the subject does not identify an influence of prenatal diagnosis, terminations of pregnancy, selective abortions or stillbirths.

### 3.5. Results of Syntheses

A discrepancy was identified between the findings of Irl et al. [29] and Scherb and Weigelt [28]. This inconsistency may be attributed to the divergent methodologies used in these studies.

**Table 3:** Results of the individual studies, part 1.

Author & Year of Publication	Period of Investigation	Place of Investigation	Used Method (Further Specified in Table 4)	Outcomes (Further Specified in Table 5)	Missing Data/Limitations
Scherb and Weigelt (2004) [28]	1984–1991	Free State of Bavaria	A trend analysis was conducted to ascertain the prevalence of the condition in each district and independent city in Bavaria, which numbered 96 in total. This analysis was then compared with the area contamination values (Cs-137). The number of surplus CL/P cases was calculated using a change point model. Finally, a comparison was made between the collected data and that of the GDR cleft register.	During the study period, 1324 patients with CL/P were recorded. A one-sided significance (*p* = 0.10) with the prevalence increase of 9.5% was observed between October 1986 and December 1990. The average increase in cleft lip and palate cases in the three most affected districts after 1986 is approximately 70%. Using the change-point model, it was calculated that 65–80 additional cases of CL/P occurred between October 1986 and December 1990 due to increased Cs-137 concentrations.	The analysis lacks statistical rigor, as the statistical values were not consistently presented in their entirety.
Irl et al. (1995) [29]	1984–1987	Free State of Bavaria	The data was collected from the birth registers of Bavarian children’s hospitals and those of the adjacent border hospitals of Ulm and Salzburg. The Cs-134 and Cs-137 were used as indicators of radiation exposure. Thereafter, the Bavarian administrative districts were divided into two categories: those with higher (southern Bavarian) and those with lower (northern Bavarian) contamination. Odds-ratio (regarding CLP and CP) was calculated for southern and northern Bavaria during designated timeframes set by the author (see Table 4 and Table 5) and later tested for significant differences.	During the study period, 330 patients with CLP and 165 with CP were recorded. The study cites an average additional radiation exposure of 0.2 mSv for the area north of the Danube, 0.6 mSv for the area south of the Danube and 1.2 mSv for the Alpine foothills. A comparative analysis of the prevalence in Northern and Southern Bavaria regions did not reveal any statistically significant differences. A comparison of the entire period reveals that 189 children with CLP and 84 with CP were born in southern Bavaria. In the northern region of Bavaria, the figures were 141 and 81, respectively.	The division of Bavarian administrative districts according to soil contamination with Cs-isotopes is based on mean values with high standard deviations. Other possible confounding factors, such as variations in terrestrial radiation by other radioactive nuclides, were not taken into account.
Zieglowski and Hemprich (1999) [27]	1970–1989	Former GDR	Evaluation of the GDR malformation registry for CL/P between 1980–1989. Contamination data (Levels of Cs-137 and Sr-90) was compared to the prevalence within 13 of the 15 districts. This data, however, does not seem to be included in the statistical analysis of the prevalence. Further information would be needed.	In the years 1983, 1987 and 1988, the prevalence was determined to be above the confidence interval. In 1987, a 9.4% increase in the prevalence of children with CL/P was observed in 13 out of the 15 districts of the GDR, with data unavailable for two districts. In January 1987, a 30.1% rise in prevalence was documented compared to the same month in the preceding year. In February, the increase was 22%, and in March, it was 17.9%. A notable observation is that the average increase was highest in the GDR districts where the highest levels of Cs-137 and Sr-90 were measured.	Given that the total number of meteorological measuring stations in the former GDR at which ground-level radioactivity was measured was 8, it is impossible to link the epidemiological data on CL/P directly to the contamination, as the data is too inaccurate.
Rösche et al. (1998) [26]	1983–1995	Magdeburg area	The data collated was obtained from the Magdeburg Malformation Registry. The annual prevalence of cleft lip and palate, including associated malformations, was considered in the study.	During the study period, 309 patients with CL/P were recorded. For CL/P (including Pierre-Robin-Sequence; PR), statistical significance was found in the increasing birth prevalence per 10,000 births in the Magdeburg area in 1988, 1989, and 1995	The authors of the study refer to disturbing factors; however, they state that further consideration of these factors is not possible due to insufficient data. Dosimetry is not delineated within the methodology.
Schlemme (1996) [31]	1984–1993	Ruhr region (North Rhine-Westphalia)	A comprehensive analysis was conducted on data retrieved from the maternity wards of 34 hospitals situated within 10 randomly selected cities in the Ruhr region. A distinction was made between isolated cleft formations, cleft formations with accompanying malformations, and cleft formations associated with syndromes.	During the study period, 291 patients with CL/P were recorded. “In general, no increase or decrease in the incidence of malformations could be documented between 1984 and 1993.” The most notable increase occurred in 1989, while the two years prior and subsequent demonstrated remarkably low figures. In 1987, the proportion of children with CL/P among all recorded live births in the Ruhr region was 0.118%.	Documentation errors in the hospitals could account for a proportion of the variation. Dosimetry was not a component of the study design. The data set on stillbirths is incomplete. Furthermore, in this context, the documentation encompasses those malformations that are presumed to have resulted in fatal outcomes. Consequently, the findings allow for the formulation of only limited conclusions concerning the proportion of stillbirths accompanied by CL/P.
Petersen (2002) [30]	1974–1998	Cleft centre of the central hospital in Free Hanseatic City of Bremen	The data was collected retrospectively using patient documentation forms. The study’s approach incorporated a range of variables, including the prevalence of cleft patients, the distribution of patients by gender, the geographical distribution of patients within their respective catchment areas, the types of clefts present, and the family and pregnancy history of the patients. The arithmetic mean and standard deviation were calculated. The *p*-value was defined as 0.05.	During the study period, 1763 patients with CL/P were recorded. An increase in the number of cleft patients was documented between 1986 and 1997 for male patients, and between 1984 and 1998 for female patients. A total of 84.99% of the cleft patients for whom geographical origin was known hailed from Bremen or Lower Saxony. In comparison with the entire study group, patients with cleft palates and right-sided CL/P were more likely to have a history of conspicuous pregnancies.	In 49.01% of the cases reviewed, a documented pregnancy history was not available. This limitation hinders the investigation of the potential influence of confounding factors. Dosimetry has not been conducted.

**Table 4:** Results of the individual studies, part 2.

Quality of Dosimetry	Publication	Outcome/Population	Exposure Measurement	Comparator	Statistical Method	Measured Confounder	Assessment
Best relative exposure assessment. Dosimetry at regional level.	Scherb and Weigelt (2004) [28]	CL/P in Bavaria. Malformation records from 1984 to 1991. 1324 cases of CL/P among 984,570 births.	Cs-137 surface contamination at district level. No individual dose estimates.	Trends before and after the Chernobyl NPP accident and a spatio-temporal comparison based on contamination levels by district. Additionally, a comparison with GDR data from 1984 to 1989.	Spatio-temporal ecological analysis. Relative risk per kBq/m^2^. Trend analysis.	No individual confounders.	The strongest of the six studies for an exposure response hypothesis, but still based on ecological evidence. The dose–response relationship cannot be demonstrated on an individual basis as it was not measured.
Average exposure assessment. Contamination groups	Irl et al. (1995) [29]	Bavaria. Isolated congenital malformations, Down’s syndrome. Mortality up to the age of one. Children born between 1984 and 1987. Mortality rates between 1980 and 1992. During the study period, 330 patients with CLP and 165 with CP were recorded among 381,074 births.	Cs-134/Cs-137 in soil samples. 1137 Bavarian sites. 8 km grid. Classified as southern and northern Bavaria or higher/lower contamination.	Regions with higher levels of contamination in southern Bavaria were compared with regions of lower levels of contamination in northern Bavaria. Additionally, the period following the Chernobyl NPP accident was compared with the corresponding prior period.	Odds ratios: Southern vs. Northern Bavaria Comparison of observed/expected mortality. *p*-values, α-adjustment according to Horn for multiple tests. (CI: 95%; *p*-value: 0.05)	No individual confounders.	No significant increase in severe malformations or mortality. Methodologically superior to simple before-and-after studies, but based solely on ecological exposure.
Widespread regional exposure. Weak methodical dosimetry	Zieglowski and Hemprich (1999) [27]	Former GDR. Central CL/P register between 1980 and 1989. Cases of CL/P per 1000 live births. Both the number of births and cases of CL/P are not available.	Environmental data from national and international reports. The GDR monitoring network comprised only eight stations and γ-spectrometric single-nuclide analysis was carried out only at Berlin-Friedrichshagen.	The pre-Chernobyl period, particularly 1980 to 1986 was compared to the post Chernobyl year of 1987. Additionally, a regional analysis of the districts of the GDR, with a focus on the northern districts, which had higher Cs-137/Sr-90 levels.	Comparison with a 95% CI and a significance level of *p* = 0.05 Regional description.	No individual confounders.	There is evidence of an increased prevalence in certain periods or regions, but the exposure data is too imprecise. The data provided is too little to show a dose–response relationship.
No dosimetry. Before and after trend.	Rösche et al. (1998) [26]	Magdeburg region between 1983 and 1995. 171,182 births. 309 children with CL/P.	Not performed.	A comparison over time within the Magdeburg region, focusing primarily on the years before and after 1986.	Comparison of prevalence over the years. Statistical significance (CI: 95%; *p*-value: 0.05) reported.	No individual confounders.	Useful for describing prevalence. Weak for establishing causality in the case of the Chernobyl NPP accident as there is no dosimetry.
No dosimetry. Regional incidence study	Schlemme (1996) [31]	Ruhr region between 1984 and 1993. 236,357 live births. 291 cases of CL/P.	Not performed.	Year-on-year and city-to-city comparisons within the Ruhr region.	95% CI according to Blyth and Still to presume the actual amount of cases of CL/P within each city using data from part of their hospitals. Descriptive comparisons between cities and years.	No individual confounders. However, the author explicitly clarifies that such factors such as environmental pollution, demographic structure, social status, the proportion of foreign residents, health awareness, etc., are unknown.	Good raw figures, but no exposure measurements. Of very limited use in the context of the Chernobyl NPP accident
No dosimetry. Clinical case series.	Petersen (2002) [30]	Bremen/Lower Saxony. Cleft centre of the central hospital in Free Hanseatic City of Bremen between 1974 and 1998. 1763 patients of whom 1737 had a precise diagnosis.	Not performed.	Trends over time with a clinical cohort.		No individual confounders.	Hardly suitable for aetiology/Chernobyl, as there is no common denominator, no dosimetry and no exposure model. It is more of a clinical and epidemiological review.

**Table 5:** Results of the individual studies, part 3.

Publication	Pre-/Post-Chernobyl Numbers
Scherb and Weigelt (2004) [28]	Oct. 1986–Dec. 1990 +9.5% increase in cases with CL/P. *p* = 0.10. Correlation: RR per kBq/m^2^ = 1.008, *p* = 0.03. Bavaria and the GDR combined, 1984–1989: increase in cases with CL/P of +8.6%, *p* = 0.02. Cases of CL/P: 1984: 148/111,183 = 1.33/1000 1985: 142/111,365 = 1.28/1000 1986 (Jan–Sep.): 116/89,690 = 1.29/1000 1987(Oct. 86–Dec.87): 203/148,372 = 1.37/1000 1988: 174/126,409 = 1.38/1000 1989: 183/127,029 = 1.44/1000 1990: 193/136,122 = 1.42/1000 1991: 165/134,400 = 1.34/1000
Irl et al. (1995) [29]	cases of CLP and CP among births: Jan. 1984–Apr. 1986: Southern Bavaria (SB) 131 (CLP) and 59 (CP)/146,557, Northern Bavaria (NB) 90 (CLP) and 56 (CP)/114,624. Dec. 1986–Feb. 1987: SB 11 (CLP) and 6 (CP)/16,164, NB 10 (CLP) and 7 (CP)/12,473. No significant difference can be found between the odds ratio in SB and NB (*p*-value: 0.51 (CLP), 0.71 (CP)) March–May 1987: SB 11 (CLP) and 9 (CP)/16,849, NB 13 (CLP) and 3 (CP)/13,155. No significant difference can be found between the odds ratio in SB and NB (*p*-value: 0.21 (CLP), 0.15 (CP)) June–August 1987: SB 18 (CLP) and 6 (CP)/16,849, NB 17 (CLP) and 10 (CP)/13,155. No significant difference can be found between the odds ratio in SB and NB (*p*-value: 0.38 (CLP), 0.32 (CP)) September–November 1987: SB 18 (CLP) and 4 (CP)/16,849, NB 11 (CLP) and 5 (CP)/13,155. No significant difference can be found between the odds ratio in SB and NB (*p*-value: 0.81 (CLP), 0.69 (CP)) None of the designated timeframes showed significant (*p*-value ≤ 0.05) differences in prevalence between NB and SB for CLP and CP.
Zieglowski and Hemprich (1999) [27]	1980–1989 prevalence of CL/P Significant increases in the prevalence of CL/P found in 1983, 1987 and 1988 average rate 1.88/1000, 95% CI 1.80–1.95/1000. Pre-1980–1986: 1.828/1000; 1987: 2.000/1000, +9.4% increase in prevalence. Northern districts: Schwerin +45.5%, Neubrandenburg +39.6%, Rostock +34.8%. *Exact p-values for the reported significant annual increases were not reported in the source.*
Rösche et al. (1998) [26]	prevalence 18.05 per 10,000 live births. Significant increases in prevalence of CLP, CP and Pierre-Robin-Sequence (PR) associated clefts combined in 1988, 1989 and 1995. 22.86 per 10,000 (1988) 25.65 per 10,000 (1989) 22.07 per 10,000 (1995) with an overall prevalence of 18.05 per 10,000 (1980–1989). (CI: 16.18–20.12) 1985, 1987 and 1992 also find a significant difference. Those values were, however, below the CI. 13.40 per 10,000 (1985) 14.56 per 10,000 (1987) 14.41 per 10,000 (1992) (CI: 16.18–20.12) CLP: Prevalence per 10,000 Above CI: 1988 (19.25), 1989 (16.45), 1994 (14.7). Below CI: 1984 (9.22), 1985 (8.16), 1987 (9.9), 1992 (10.48). (CI: 11.08–14.36) CP: Prevalence per 10,000 Above CI: 1989 (9.21), 1993 (5.79), 1995 (7.79). Below CI: 1983 (2.29), 1988 (3.01), 1990 (3.44), 1991 (1.1) (CI: 3.6–5.69) CLP and CP are differentiated. Exact numbers for PR are not specified. 67 out of 309 cases demonstrated additional malformations. *Exact p-values for the reported significant annual differences were not reported in the source.*
Schlemme (1996) [31]	Total CL/P cases: 291/246,357 = 0.118% = 1.18/1000. 1984–1986: 65/64,904 ≈ 1.00/1000. (1984: 19; 1985: 24; 1986: 22) 1987–1989: 93/75,004 ≈ 1.24/1000. (1987: 28; 1988: 21; 1989: 44) Year 1989: 44/25,897 = 1.70/1000. (A quarter of these cases were documented in Duisburg in 1989. Whereas in 1988 there had only been 3 cases in Duisburg. At the same Time, no cases were documented in Bochum and Dortmund during 1989.) 1990–1993: 133/106,449 = 1,25/1000 (1990: 27; 1991: 38; 1992: 35; 1993: 33) 19 (6.53%) clefts were associated with some kind of syndrome. 7 out of these 19 were shown to have a PR-Sequence. In total 81.1% out of 291 cases were identified as isolated clefts. 12.37% either died within the first month or showed additional malformations. The author does not differentiate different types of clefts or their association with syndromes or other malformations on a yearly basis. *No formal before-and-after significance analysis was reported in the source.*
Petersen (2002) [30]	No prevalence, as there is no population-based denominator. Cases of CL/P: 1980: 43; 1981: 40; 1982: 53; 1983: 53; 1984: 68; 1985: 59; 1986: 78; 1987: 89; 1988: 74; 1989: 88; 1990: 83; 1991: 98; 1992: 93; 1993: 101; 1994: 94; 1995: 88. cases of CP: 1980: 12; 1981: 8; 1982: 13; 1983: 19; 1984: 17; 1985: 27; 1986: 26; 1987: 36; 1988: 31; 1989: 31; 1990: 25; 1991: 27; 1992: 29; 1993: 32; 1994: 24; 1995: 34. 273 (15.48%) syndromic cases were documented. 143 (8.11%) of which were diagnosed as PR. 67.61% cases diagnosed as isolated clefts. The author does not differentiate the association with syndromes or other malformations over the years. *No formal before-and-after significance analysis was reported in the source.*

Abbreviations (Table 3, Table 4 and Table 5): PR, Pierre-Robin-Sequence. CL/P, cleft lip and/or palate. CLP, cleft lip and palate. CP, cleft palate. GDR, German Democratic Republic. Bq, becquerel. Sv, sievert. Cs, caesium. Sr, Strontium. CI, confidence interval. SB, southern Bavaria. NB, northern Bavaria. NPP, nuclear power plant.

As previously elucidated, Irl et al. [29] delineate the term Northern Bavaria as encompassing the Upper Palatinate, Upper Franconia, Middle Franconia, and Lower Franconia. Southern Bavaria includes the administrative districts of Upper Bavaria, Lower Bavaria, and Swabia. While Scherb and Weigelt [28] determined the dosage for each of the 96 districts, Irl et al. [29] determined only the dosage for each of the seven counties to which the individual districts were subordinate. The assertion was that “Southern Bavaria” experienced higher levels of contamination than Northern Bavaria. The average decay rates in Southern Bavaria were approximately 20 kBq/m^2^ higher than those in Northern Bavaria. For example, an average value of 34 kBq/m^2^ was reported for Swabia [29]. Augsburg is in Swabia. Scherb and Weigelt [28] calculated an average decay rate of 53.7 kBq/m^2^ for Augsburg (city). This value is nearly 20 kBq/m^2^ higher than that calculated for Swabia by Irl et al. [29]. This indicates that values below and above 34 kBq/m^2^ were likely highly dispersed, implying substantial regional heterogeneity. Irl et al. [29] described contamination in Germany as extremely inhomogeneous. The choice of a methodology that homogenizes contamination in this manner is questionable. The situation in Upper Bavaria is analogous to that in Swabia. Berchtesgaden (50.3 kBq/m^2^) and Garmisch-Patenkirchen (40.5 kBq/m^2^) are in Upper Bavaria. Irl et al. [29] give a value of 32 kBq/m^2^ for Upper Bavaria. The data revealed that none of the three most contaminated districts or independent cities were located in Lower Bavaria. Although Lower Bavaria exhibited a higher level of contamination (22 kBq/m^2^ [29]) than the other districts in Northern Bavaria, it was less contaminated than the districts of Upper Bavaria and Swabia in Southern Bavaria. This may have distorted the regional comparison.

### 3.6. Certainty of Evidence

Table 6 and Table 7 show the certainty of evidence for the considered outcome. Table 6 provides study-level information, whereas Table 7 summarises the overall certainty assessment.

The certainty of the evidence was downgraded because the given factors were applied. None of the factors related to upgrading could be applied. The risk of bias was probably high in three out of six studies, mostly due to a high risk of detection and confounding bias, several cases of selective reporting, and failure to declare conflicts of interest. The other three studies, deemed probably low, did not explain how dosimetry was performed or did not perform it at all.

There were inconsistencies between the studies. Some studies did not directly answer whether the incidence of CL/P changed. Imprecision was high owing to the large confidence intervals, and residual confounding factors were ignored in all the studies involved.

It is important to emphasise that the dose–response gradient remains to be clarified. Weinberg et al. [32] suggested 50–200 mGy exposure was needed to induce malformations. Petrova’s work also cites this dose [33]. It is unclear whether individual doses reached this level; however, this is unlikely, as Irl et al. [29] reported that the mean additional dose due to Chernobyl in the most strongly contaminated areas of Germany was approximately 1.2 mGy. A meta-analysis conducted in 2024 did not provide a clear answer on this topic [34]. Another meta-analysis considered the influence of ionising radiation, even below 100 mGy [35].

The discussions held with and the written correspondence exchanged between researchers during this investigation also indicated that there are a considerable number of unfinished or never-published scientific papers on this topic, suggesting publication bias.

## 4. Discussion

### 4.1. Interpretation

To the best of our knowledge, this is the first systematic review to consider the influence of ionising radiation from the Chernobyl NPP accident on CL/P formation in Germany. Because the included studies were heterogeneous in design, exposure assessment, outcome definition, and statistical reporting, the Bradford Hill criteria were used as a structured interpretative framework to assess the plausibility of causal evidence. These criteria were not applied as a formal proof of causality and were not interpreted as a checklist with a required minimum number of fulfilled domains. Rather, they were used to organise the available evidence according to established causal considerations, including strength of association, consistency, temporality, biological gradient, plausibility, coherence, experiment, and analogy, where applicable. This approach was considered suitable for a sparse and heterogeneous evidence base in which quantitative pooling was not methodologically appropriate. The criteria under consideration comprise nine distinct domains.

The first domain is strength of association. A very low *p*-value may indicate a strong association in a sufficiently powered analysis [36]; however, this was not consistently evident across the included studies.

Second, the requirement for consistency must be observed. The areas of particular interest—those that demonstrated particularly elevated levels of contamination—exhibited an absence of consistency in the study results in relation to CL/P prevalence [28,29].

To comply with the third domain, it is necessary to ascertain the specificity. As previously stated, several confounding factors should be considered. It is plausible that these are indistinguishable from the ionising radiation in Germany following the Chernobyl NPP accident. In addition to ionising radiation, maternal obesity, active or passive smoking, and type 1 diabetes have also been identified as risk factors for CL/P [37]. Moreover, a separate meta-analysis identified maternal drug intake, socioeconomic status, and several genes as risks [38].

The fourth domain is temporality. Some studies observed fluctuations in CL/P incidence even before the Chernobyl NPP accident. This is important because such temporal variation may also occur independently of radioactive fallout. Scherb and Weigelt [28] described comparable fluctuations, although these were not consistently reported as statistically significant.

The fifth domain is biological gradient. The dose–response relationship remains ambiguous. Despite considerable research effort, the existing literature has not provided a conclusive answer [34,35]. Animal studies using substantially higher exposures have demonstrated more pronounced effects on CL/P prevalence. In one study, pregnant mice were exposed to 4 Gy of radiation on gestational day 11.5; of 32 offspring, 27 (84.4%) exhibited signs of CL/P [39]. In another study, mice were irradiated on gestational day 12 at a dose of 4 Gy, and 91% of foetuses exhibited CL/P [40]. A further study reported similar outcomes at different gestational days, although prevalence varied [41].

The sixth domain is plausibility. The precise underlying mechanism of CL/P remains unclear. The effect of ionising radiation was stochastic. A search of the PubMed database yielded no results on this subject. Damage to deoxyribonucleic acid can result in congenital malformations. A genetic effect on CL/P has been attributed to *MTHFD1* 1958G and *MTHFR* c.677C [42]. Recent research has identified more than 60 risk loci [43].

The seventh domain is coherence. Coherence between laboratory and epidemiological findings is limited because the epidemiological findings are inconsistent. Although ionising radiation has been shown to induce CL/P in experimental models, this effect has been observed only in murine studies at doses that are disproportionate to those associated with the Chernobyl NPP accident in Germany.

The eighth domain concerns experimental evidence. Human experimental studies approximating the exposure scenario are not ethically possible; therefore, most experimental evidence derives from animal studies.

Finally, analogy was considered. This domain cannot be fully satisfied, but comparable scenarios involving the release of radioactive particles provide useful context. One analogy is the aftermath of the Fukushima nuclear disaster; both Fukushima and Chernobyl were classified as Level 7 events on the International Nuclear Event Scale, although the quantity of radioactive material released during the Chernobyl NPP accident was considerably higher. A study conducted in Japan found no substantial changes in the incidence of congenital malformations [44]. Engeln and Pausch [25] examined the influence of radon on birth rate and CP prevalence in 13 of 15 districts in the GDR between 1980 and 1989. As in Zieglowski and Hemprich [27], the data were obtained from the official GDR malformation register. The authors concluded that, compared with the group exposed to less than 69 kBq/m^3^, a significantly higher number of children with CP were born in the group exposed to an average of more than 69 kBq/m^3^.

The results of the Bradford-Hill analysis suggest that the currently available evidence does not strongly support a causal relationship. However, this methodological assessment does not constitute proof of the absence of a causal relationship. Three studies demonstrated a correlation in terms of statistically significant changes in the temporal context. It is important to acknowledge that Hill himself recognised the provisional nature of scientific work, stating, “all scientific work is incomplete… [and] liable to be upset or modified by advancing knowledge” [45].

The linear-no-threshold (LNT) model has primarily been developed and applied in radiation risk assessment for cancer and is not an established framework for congenital malformations [46,47,48,49,50]. We therefore do not use LNT to interpret the CL/P findings in this review. The available literature did not provide sufficient evidence to evaluate an LNT-type dose–response relationship for malformations. Although an influence in Germany cannot be ruled out with certainty, substantiating such a claim is challenging. The profound impact of the Chernobyl NPP accident on affected populations, particularly in Belarus and Ukraine, should nevertheless not be disregarded [11,51,52]. Germany was represented only partially in the available evidence; the included studies covered Bavaria, the GDR, parts of the Ruhr region, and Bremen. The dataset therefore lacks important information. Moreover, the evidence is difficult to evaluate because potential conflicts of interest and funding sources were often not reported, and several studies lacked key statistical elements such as confidence intervals, standard deviations, or *p*-values, even when other figures were provided in the same study.

The question of long-term effects remains difficult to answer. Given the half-life of Cs-137, contamination levels would be expected to have decreased substantially over time, although decay products may also be radioactive. Several studies have examined CL/P prevalence after the Chernobyl NPP accident [15,16,17,53]. However, the hypothesis that later increases in CL/P prevalence are attributable to Chernobyl cannot be distinguished from other risk factors for CL/P. In view of the low radiation exposure in Germany and the presence of other potential influencing factors, the authors of this review consider the probability of such an effect to be low.

### 4.2. Limitations of Evidence

Precise individual dosimetry is particularly challenging at the population level in a country the size of Germany.

Second, the aetiology of CL/P remains an area of enquiry that has not yet been fully elucidated. This includes the fact that the influence of radioactivity remains an open question that requires further research [54,55,56].

Third, the data collection was predominantly retrospective. This methodological approach is vulnerable to inaccuracies, which may have contributed to the potential errors observed in the studies conducted by Irl et al. [29] and Scherb and Weigelt [28], resulting in divergent outcomes. A notable limitation of these studies is the possibility that a significant number of patients may not have received treatment at children’s hospitals, resulting in their exclusion from the data collection. Scherb and Weigelt [28] posited that the baseline prevalence of CL/P in the GDR was 43% higher. However, given that both the GDR and Bavaria belong to a similar ethnic group, it can be assumed that a considerable number of cases remain unrecorded. A formal registry was operational within the GDR, wherein all cases were documented at the time of birth.

### 4.3. Limitations of Review Processes

Although online and library-based searches were undertaken, the review may not have identified all relevant literature. Scopus and ScienceDirect were not searched as part of the original strategy, and grey literature was not systematically searched beyond the two dissertations identified through the library search; these choices may have reduced retrieval breadth. Some older papers may not yet have been digitised, some studies may have been written but never published, and some projects may have been discontinued before analysis or publication. Locating sources cited in older bibliographies was a recurrent challenge, and only one such source could ultimately be obtained.

Furthermore, effective communication with researchers was hindered because a significant proportion of those who have studied this subject are now retired.

The search strategy did not undergo formal peer review, and alternative databases or search formulations might retrieve additional records. These limitations should be considered when interpreting the completeness of the evidence base.

### 4.4. Implications

A small body of evidence indicates the compelling need to further investigate the consequences of low-dose radiation exposure. Therefore, it is imperative to conduct research in this area. In the future, these events should be used for research purposes, given the ethical prohibitions on laboratory studies involving ionising radiation in humans. Considering the prominence of Chernobyl, there is an evident incongruence between the quantity and quality of available research.

In the context of the present-day Federal Republic of Germany, the establishment of a comprehensive and uniform malformation registry is imperative. Such an initiative should incorporate standardised data collection procedures to minimise distortions arising from misclassification or miscommunication.

The authors acknowledge the contributions of EUROCAT. This uniform European registry records a wide range of malformations, including CL/P. However, at present, only the Saxony-Anhalt malformations registry participates in EUROCAT for Germany [57]. This constitutes a segment of the data analysed by Rösche et al. [26]. EUROCAT data available for Germany are limited.

The need for a better registry is not limited to the Chernobyl context. The Chernobyl NPP accident is now a historical event, and other factors can also alter the prevalence of malformations. For example, the thalidomide scandal highlighted the importance of a uniform malformation registry. Such a registry may have facilitated earlier identification of associated dangers. Thalidomide, the active substance in Contergan, has been shown to induce severe congenital anomalies [58,59].

## 5. Conclusions

Considering the limitations of the present review, some included studies reported associations between the Chernobyl accident and CL/P prevalence in more heavily contaminated areas of Germany, including parts of Bavaria and the former GDR, whereas no statistically significant differences were found in regions affected by lower levels of fallout. However, the evidence base is small, heterogeneous, and of very low certainty. Exposure was assessed ecologically rather than at the individual level, several studies had important methodological and reporting limitations, and confounding factors were not adequately controlled. Accordingly, the available evidence does not support establishing a causal relationship between Chernobyl-related exposure and CL/P in Germany. The literature also distinguishes incompletely between cleft subtypes and between syndromic and non-syndromic cases, while the dose–response relationship remains uncertain. One study estimated an additional regional exposure of approximately 1.2 mGy in the most heavily contaminated areas of Bavaria; this value does not replace individual dosimetry and should be interpreted cautiously. Several cited sources discuss malformation effects at doses of at least 50 mGy, but these values are not directly comparable with the regional estimate. The observed regional associations should therefore not be interpreted as evidence of causality. The limited quantity and quality of the available evidence nevertheless warrant further research. Future studies would benefit from a uniform cleft registry for Germany, improved exposure assessment, systematic control of confounding, and clearer distinction between cleft phenotypes.

## Figures and Tables

**Table 1:** Four-field table of the included studies.

Records Included	Significant Change in Prevalence	No significant Change in Prevalence
More severely contaminated areas	Rösche et al. [26] Zieglowski and Hemprich [27] Scherb and Weigelt [28]	Irl et al. [29]
Less severely contaminated areas		Petersen [30] Schlemme [31]

**Table 2:** Risk of Bias evaluation.

RoB-Assessment	Petersen [30]	Rösche et al. [26]	Scherb and Weigelt [28]	Schlemme [31]	Irl et al. [29]	Zieglowski and Hemprich [27]
Selection	−	−	−	−	+	−−
Confounding		+	+		+	+
Attrition/exclusion	+	−−	−	−−	−	−−
Detection		++	+		++	++
Selective reporting	−−	+	+	−	+	−
Other sources of bias	−−	NR	NR	−−	NR	NR
Final judgement	−(1.75)	+(2.7)	+(2.7)	−(1.5)	+(3)	−(2.3)

+ for “probably high”, ++ for “definitely high”, − for “probably low”, and −− for “definitely low”.

**Table 6:** Individual GRADE-Certainty-Assessment.

Publication	Risk of Bias	Inconsistency	Indirectness	Imprecision	Reasoning for Downgrading	GRADE Certainty
Scherb and Weigelt (2004) [28]	Probably high (+)	Serious/unclear: Different analyses do not yield consistent levels of evidence. The temporal effect in Bavaria is only marginally significant, whilst the spatio-temporal model is significant.	Serious: Exposure is limited to environmental contamination via soil.	Serious: Small effect per kBq/m^2^. No full confidence intervals in the table summary. *p*: 0.10 for the main time frame in Bavaria.	Downgrade due to RoB-Assessment, indirectness and imprecision. No upgrade despite the dose gradient, as confounding cannot be ruled out.	Very low
Irl et al. (1995) [29]	Probably high (+)	Not serious: The results within the study were consistent with a null hypothesis or were not statistically significant.	Serious: Regional contamination category rather than individual dose. Misclassification due to change in residence cannot be determined.	Serious: Many rare outcomes and short exposure windows. Many effect estimates presented only as ORs/*p*-values without precise confidence interval estimates.	Downgrade due to RoB-Assessment, indirectness and imprecision. No upgrading factor could be applied.	Very low
Zieglowski and Hemprich (1999) [27]	Probably low (-)	Serious: There was also a significant increase in 1983, i.e., before the Chernobyl NPP accident	Very serious: Regional radioactivity data in the GDR were insufficient (Table 3 and Table 4).	Serious: Effect reported as a percentage increase in prevalence. No model-based adjusted effect measures with CIs for exposure-outcome relationship.	Downgrade for inconsistency, strong indirectness and imprecision. No upgrading factor could be applied.	Very low
Rösche et al. (1998) [26]	Probably high (+)	Serious/unclear: Peaks in prevalence in 1988/1989, but no proper exposure-time modelling. The signal cannot be clearly distinguished from the background trend or detection variation.	Very serious: No measured exposure. No regional dosimetry in the model. Exogenous influences are merely discussed.	Serious: Relatively few cases for year-specific increases. No adjusted effect sizes or CIs for an association with the Chernobyl NPP accident	Downgrade due to RoB-Assessment, inconsistency, very strong indirectness and imprecision. No upgrading factor could be applied.	Very low
Schlemme (1996) [31]	Probably low (-)	Not serious/unclear: No consistent upward trend was observed in the study. The 1989 peak was not replicated across the board in all cities and mostly visible in Duisburg.	Very serious: No measured exposure. No regional dosimetry in the model. Exogenous influences are merely discussed.	Serious: No measurement of exposure. No exposure-outcome study design.	Downgrade due to very strong indirectness and imprecision. No upgrading factor could be applied.	Very low
Petersen (2002) [30]	Probably low (-)	Not assessable: No proper exposure analysis. Therefore, it is not possible to assess the consistency of any radiation effect.	Very serious: No dosimetry. No population-based denominator.	Very serious: No measurement of exposure. No population-based denominator.	Downgrade due to very strong indirectness and very strong imprecision. No upgrading factor could be applied.	Very low

Abbreviations: RoB, risk of bias. Bq, Becquerel. NPP, nuclear power plant. OR, Odds ratio. CI, confidence interval.

**Table 7:** General GRADE-Certainty-Assessment.

Outcome	Number of Studies	Certainty	Comment
Change in Prevalence of Orofacial clefts (CL/P; CLP; CP) post Chernobyl NPP accident	6	Very low	Missing individual dosimetry/exposure measurement, substituted by indirect environmental data. Missing confounder analysis and therefore no effect adjustment. Effects were measured even before the Chernobyl NPP accident. Sample size was too small, as CL/P is a rare outcome.

Abbreviations: CL/P, cleft lip and/or palate. CLP, cleft lip and palate. CP, cleft palate. According to [14].

## Data Availability

All data supporting the findings of this study are available within the article. Additional de-identified data may be made available by the corresponding author upon reasonable request.

## Supplementary Materials

The following supporting information can be downloaded at: https://www.mdpi.com/article/10.3390/epidemiologia7050123/s1, Table S1: PRISMA 2020 statement and checklist.

## References

[1] Strahlenschutzkommission (2006). 20 Jahre Nach Tschernobyl—Eine Bilanz aus Sicht Des Strahlenschutzes. https://ssk.de/SharedDocs/Beratungsergebnisse_PDF/2006/20JahreTschernobyl.pdf.

[2] (2025). Gesundheitliche Folgen des Unfalls von Tschornobyl in Deutschland und Europa Außerhalb der Ehemaligen Sowjetunion Bundesamt Für Strahlenschutz. BfS. https://www.bfs.de/DE/themen/ion/notfallschutz/notfall/tschornobyl/gesundheitsfolgen-europa.html.

[3] International Physicians for the Prevention of Nuclear War German Section (2006). Gesundheitliche Folgen von Tschernobyl: 20 Jahre nach der Reaktorkatastrophe.

[4] BfS, Tschornobyl (Tschernobyl)—Gesundheitliche Folgen des Unfalls von Tschornobyl in Deutschland und Europa Außerhalb der Ehemaligen Sowjetunion (2024), Web. Archive.org. https://web.archive.org/web/20240426192918/https:/www.bfs.de/DE/themen/ion/notfallschutz/notfall/tschornobyl/gesundheitsfolgen-europa.html.

[5] Marino F., Nunziata L. (2018). Long-term consequences of the Chernobyl radioactive fallout: An exploration of the aggregate data. Milbank Q..

[6] Dolk H., Nichols R. (1999). Evaluation of the impact of Chernobyl on the prevalence of congenital anomalies in 16 regions of Europe. EUROCAT Working Group. Int. J. Epidemiol..

[7] Körblein A., Küchenhoff H. (1997). Perinatal mortality in Germany following the Chernobyl accident. Radiat. Environ. Biophys..

[8] Sperling K., Pelz J., Wegner R.D., Schulzke I., Struck E. (1991). Frequency of trisomy 21 in Germany before and after the Chernobyl accident. Biomed. Pharmacother..

[9] Grosche B., Irl C., Schoetzau A., van Santen E. (1997). Perinatal mortality in Bavaria, Germany, after the Chernobyl NPP accident. Radiat. Environ. Biophys..

[10] Little J. (1993). The Chernobyl accident, congenital anomalies and other reproductive outcomes. Paediatr. Perinat. Epidemiol..

[11] Cardis E., Hatch M. (2011). The Chernobyl accident—An epidemiological perspective. Clin. Oncol..

[12] Page M.J., McKenzie J.E., Bossuyt P.M., Boutron I., Hoffmann T.C., Mulrow C.D., Shamseer L., Tetzlaff J.M., Akl E.A., Brennan S.E. (2021). The PRISMA 2020 statement: An updated guideline for reporting systematic reviews. BMJ.

[13] National Toxicology Program (NTP) (2015). OHAT Risk of Bias Rating Tool for Human and Animal Studies.

[14] Schünemann H.J., Brennan S., Akl E.A., Hultcrantz M., Alonso-Coello P., Xia J., Davoli M., Rojas M.X., Meerpohl J.J., Flottorp S. (2023). The development methods of official GRADE articles and requirements for claiming the use of GRADE—A statement by the GRADE guidance group. J. Clin. Epidemiol..

[15] Olbertz D., Voigt M., Straube S., Renz I., Steinbicker V., Pötzsch S., Briese V. (2010). Angeborene Fehlbildungen—Eine systematische Kohortenstudie aus Mecklenburg-Vorpommern. Z. Geburtshilfe Neonatol..

[16] Philipp K., Anja Q., Boris S., Johanna K., Susanne W., Adam S., Philipp M.-M., Henning S. (2023). Epidemiological and clinical evaluation of patients with a cleft in Lower Saxony Germany: A mono-center analysis. Clin. Oral Investig..

[17] Bremer S., Kiess W., Thome U., Knüpfer M., Bühligen U., Vogel M., Friedrich A., Janisch U., Rißmann A. (2018). Prävalenz von gastroschisis, omphalozele, spina bifida und orofazialen spaltbildungen bei neugeborenen im zeitraum Januar 2000–Dezember 2010 in Leipzig, Sachsen, Sachsen-Anhalt und Deutschland. Gesundheitswesen.

[18] Aghajanyan A., Kuzmina N., Sipyagyna A., Baleva L., Suskov I. (2011). Analysis of genomic instability in the offspring of fathers exposed to low doses of ionising radiation. Environ. Mol. Mutagen..

[19] Vorobtsova I.E. (2016). Before and after Chernobyl accident (memoirs, researches hypotheses). Radiats. Biol. Radioecol..

[20] Sagheri D., Ravens-Sieberer U., Braumann B., von Mackensen S. (2009). An evaluation of health-related quality of life (HRQoL) in a group of 4–7 year-old children with cleft lip and palate. J. Orofac. Orthop..

[21] Härtel J., Kriens O., Kundt G. (1991). Incidence of cleft lip, alveolus and palate forms. J. Craniomaxillofac. Surg..

[22] Krost B., Schubert J. (2006). Influence of season on prevalence of cleft lip and palate. Int. J. Oral Maxillofac. Surg..

[23] Afanasyev D.E., Liubarets S.F. (2020). Odontological effects of ionising radiation (review). Probl. Radiat. Med. Radiobiol..

[24] Bazyka D., Finch S.C., Ilienko I.M., Lyaskivska O., Dyagil I., Trotsiuk N., Gudzenko N., Chumak V.V., Walsh K.M., Wiemels J. (2017). Buccal mucosa micronuclei counts in relation to exposure to low dose-rate radiation from the Chornobyl nuclear accident and other medical and occupational radiation exposures. Environ. Health.

[25] Engeln C.A., Pausch N.C. (2025). Epidemiological analysis of the incidence of cleft lip and palate in relation to the radon concentration in the German Democratic Republic from 1980 to 1989. J. Craniomaxillofac. Surg..

[26] Rösche C., Steinbicker V., Röse I. (1998). Incidence of facial clefts in the Magdeburg region. Mund Kiefer Gesichtschir..

[27] Zieglowski V., Hemprich A. (1999). Spaltgeburtenrate der ehemaligen DDR vor und nach dem Reaktorunfall in Tschernobyl. Mund Kiefer Gesichtschir..

[28] Scherb H., Weigelt E. (2004). Cleft lip and cleft palate birth rate in Bavaria before and after the Chernobyl nuclear power plant accident. Mund Kiefer Gesichtschir..

[29] Irl C., Schoetzau A., van Santen F., Grosche B. (1995). Birth prevalence of congenital malformations in Bavaria, Germany, after the Chernobyl accident. Eur. J. Epidemiol..

[30] Petersen S. (2002). Patienten mit Lippen-, Kiefer- und Gaumenspalten: Eine Epidemiologische Studie von 1974 bis 1998. Doctoral Dissertation.

[31] Schlemme C. (1996). Neonatalstudie Über die Frequenz von Lippen-Kiefer-Gaumenspalten im Ruhrgebiet (1984–1993). Doctoral Dissertation.

[32] Weinberg H.S., Korol A.B., Kirzhner V.M., Avivi A., Fahima T., Nevo E., Shapiro S., Rennert G., Piatak O., Stepanova E.I. (2001). Very high mutation rate in offspring of Chernobyl accident liquidators. Proc. Biol. Sci..

[33] Petrova A., Nriagu J. Risk of Radiation Exposure to Children and Their Mothers. Encyclopedia of Environmental Health.

[34] Stephens J., Moorhouse A.J., Craenen K., Schroeder E., Drenos F., Anderson R. (2024). A systematic review of human evidence for the intergenerational effects of exposure to ionising radiation. Int. J. Radiat. Biol..

[35] Frangione B., Hinton P., Villeneuve P.J. (2023). Low-dose ionising radiation and adverse birth outcomes: A systematic review and meta-analysis. Int. Arch. Occup. Environ. Health.

[36] Fedak K.M., Bernal A., Capshaw Z.A., Gross S. (2015). Applying the Bradford Hill criteria in the 21st century: How data integration has changed causal inference in molecular epidemiology. Emerg. Themes Epidemiol..

[37] Ács M., Cavalcante B.G.N., Bănărescu M., Wenning A.S., Hegyi P., Szabó B., Harnos A., Gerber G., Varga G. (2024). Maternal factors increase risk of orofacial cleft: A meta-analysis. Sci. Rep..

[38] Lee K.S., Choi Y.J., Cho J., Lee H., Lee H., Park S.J., Park J.S., Hong Y.C. (2021). Environmental and genetic risk factors of congenital anomalies: An umbrella review of systematic reviews and meta-analyses. J. Korean Med. Sci..

[39] Kim S.H., Lee J.H., Oh H., Kim S.R., Lee C.-S., Jo S.K., Kim T.-H., Lee Y.-S. (2001). Dependence of malformation upon gestational age and exposed dose of gamma radiation. J. Radiat. Res..

[40] Hiranuma H., Jikko A., Maeda T., Abe M., Fuchihata H. (2000). Effect of X irradiation on secondary palate development in mice. Radiat. Res..

[41] Craenen K., Verslegers M., Buset J., Baatout S., Moons L., Benotmane M.A. (2018). A detailed characterization of congenital defects and mortality following moderate X-ray doses during neurulation. Birth Defects Res..

[42] Alghonemy W.Y., Ashmawy M.G. (2025). Meta-analysis and systematic review for the genetic basis of cleft lip and palate. J. Oral Biol. Craniofacial Res..

[43] Awotoye W., Mossey P.A., Hetmanski J.B., Gowans L.J.J., Eshete M.A., Adeyemo W.L., Alade A., Zeng E., Adamson O., Naicker T. (2022). Whole-genome sequencing reveals de-novo mutations associated with nonsyndromic cleft lip/palate. Sci. Rep..

[44] Nishigori H., Fujimori K., Hosoya M., Nishigori T., Murata T., Kyozuka H., Ogata Y., Sato A., Shinoki K., Yasumura S. (2023). Congenital anomalies in infants in Fukushima from 2011 to 2014: The Japan environment and children’s study. JMA J..

[45] Hill A.B. (1965). The environment and disease: Association or causation?. Proc. R. Soc. Med..

[46] Sutou S. (2025). Questioning the Linear No-Threshold Model (LNT): Lessons from Hiroshima/Nagasaki and Fukushima. Dose Response.

[47] Calabrese E.J., Giordano J. (2022). LNTgate: How LNT benefited from editorial actions. Chem. Biol. Interact..

[48] Calabrese E.J., Selby P.B. (2025). Embracing dishonesty: How LNT became king. Chem. Biol. Interact..

[49] Kaminski C.Y., Dattoli M., Kaminski J.M. (2020). Replacing LNT: The Integrated LNT-Hormesis Model. Dose-Response A Publ. Int. Hormesis Soc..

[50] Calabrese E.J. (2018). From Muller to mechanism: How LNT became the default model for cancer risk assessment. Environ. Pollut..

[51] Wertelecki W. (2020). Chornobyl radiation-congenital anomalies: A persisting dilemma. Congenit. Anom..

[52] Lazjuk G.I., Nikolaev D.L., Novikova I.V. (1997). Changes in registered congenital anomalies in the Republic of Belarus after the Chernobyl accident. Stem Cells.

[53] Sofi-Mahmudi A., Shamsoddin E., Khademioore S., Khazaei Y., Vahdati A., Tovani-Palone M.R. (2025). Global, regional, and national survey on burden and Quality of Care Index (QCI) of orofacial clefts: Global burden of disease systematic analysis 1990–2019. PLoS ONE.

[54] Inchingolo A.M., Fatone M.C., Malcangi G., Avantario P., Piras F., Patano A., Di Pede C., Netti A., Ciocia A.M., De Ruvo E. (2022). Modifiable risk factors of non-syndromic orofacial clefts: A systematic review. Children.

[55] Ma Q., Wei J., Peng B., Liu J., Mo S. (2025). Burden of orofacial clefts from 1990–2021 at global, regional, and national levels. Front. Pediatr..

[56] Askarian S., Gholami M., Khalili-Tanha G., Tehrani N.C., Joudi M., Khazaei M., Ferns G.A., Hassanian S.M., Avan A., Joodi M. (2023). The genetic factors contributing to the risk of cleft lip-cleft palate and their clinical utility. Oral Maxillofac. Surg..

[57] Medizinische Fakultät/Universitätsklinikum Magdeburg A. ö. R. (2017). 8. Einsendertreffen. https://www.med.ovgu.de/-p-2288.html.

[58] Vargesson N., Hooper G., Giddins G., Hunter A., Stirling P., Lam W. (2023). Thalidomide upper limb embryopathy–pathogenesis, past and present management and future considerations. J. Hand Surg. Eur. Vol..

[59] Vargesson N. (2015). Thalidomide-induced teratogenesis: History and mechanisms. Birth Defects Res. C Embryo Today.

